# Non-Syndromic Ganglioneuromatosis of the Gallbladder, an Extremely Rare Condition: Case Report and Literature Review

**DOI:** 10.3390/reports8040259

**Published:** 2025-12-07

**Authors:** Catalin-Bogdan Satala, Alina-Mihaela Gurau, Gabriela Patrichi, Daniela Mihalache

**Affiliations:** 1 Department of Morphological and Functional Sciences, “Dunărea de Jos” University of Galati, 800008 Galati, Romania; catalin.satala@ugal.ro (C.-B.S.); daniela.mihalache@ugal.ro (D.M.); 2 Department of Pathology, Clinical County Emergency Hospital Braila, 810325 Braila, Romania; 3 The School for Doctoral Studies in Biomedical Sciences, “Dunărea de Jos” University of Galați, 800008 Galati, Romania; g.alinaaa96@yahoo.com; 4 The Doctoral School of Medicine and Pharmacy, “George Emil Palade” University of Medicine, Pharmacy, Science, and Technology of Targu Mures, 540142 Targu Mures, Romania

**Keywords:** ganglioneuromatosis, gallbladder, non-syndromic

## Abstract

**Background and Clinical Significance**: Ganglioneuromatosis is a benign proliferation of mature ganglion cells, Schwann cells, and nerve fibers within the enteric or autonomic nervous system. According to the WHO classification, it encompasses a spectrum range from solitary ganglioneuroma to ganglioneuromatous polyposis and diffuse mural involvement. It is most commonly encountered in the colon and small bowel and is strongly associated with hereditary syndromes such as neurofibromatosis type 1 (NF1), multiple endocrine neoplasia type 2B (MEN2B), and Cowden syndrome. The involvement of the gallbladder is exceptional and only isolated cases have been documented. **Case Presentation**: We present the case of 64-year-old man admitted with longstanding right hypochondrium and epigastric pain, accompanied by intermittent nausea and occasional bilious vomiting. A cholecystectomy was performed and the histology result showed hypertrophic nerve fibers with interspersed mature ganglion cells within the fibromuscular layer. Immunohistochemistry supported neural origin and glial differentiations, consistent with ganglioneuromatosis of the gallbladder. The patient has no clinical evidence of NF1, MEN2B, or Cowden syndrome, adding a non-syndromic adult case to the very limited literature on this entity. **Conclusions:** This is a rare, non-syndromic adult case of gallbladder ganglioneuromatosis, contributing to the very limited literature on this entity.

## 1. Introduction and Clinical Significance

Ganglioneuromatosis of the gallbladder is an exceptionally rare and understudied entity, characterized by the benign hyperplasia of ganglion cells and nerve fibers within the fibromuscular layer of the gallbladder wall [1,2]. This condition is part of the broader spectrum of neurogenic tumors, most frequently encountered in the gastrointestinal tract. The involvement of the gallbladder is extremely uncommon. Predominantly observed in patients with multiple endocrine neoplasia 2B (MEN2B), ganglioneuromatosis of the gallbladder has also been sporadically reported in association with neurofibromatosis type 1 (von Recklinghausen disease) and Cowden syndrome [3,4,5,6,7,8].

Clinical manifestations are nonspecific and often overlap with common biliary conditions. Published cases described symptoms ranging from chronic right upper quadrant pain and biliary colic to asymptomatic lesions incidentally discovered during cholecystectomy. Radiologic findings are equally variable, frequently interpreted as chronic cholecystitis of gallbladder wall thickening, and contributing to diagnostic challenges [3,4,5,6,7,8].

To better characterize the rarity of this entity, we conducted a structured literature review across PubMed/MEDLINE, Scopus, Embase, and Web of Science, covering all records available up to November 2025. The search strategy included combinations of terms related to “ganglioneuromatosis”, “ganglioneuroma” and “neurogenic gallbladder lesion”. This review identified five documented cases of diffuse gallbladder ganglioneuromatosis, and only one of them having a non-syndromic lesion. We also found two additional reports describing a solitary polypoid gallbladder lesion subsequently diagnosed as ganglioneuroma, representing the only other gallbladder lesion with this type of differentiation published to date outside the diffuse pattern [4,5,6,7,8,9].

In this study, we report the case of a 64-year-old male diagnosed with gallbladder ganglioneuromatosis.

## 2. Case Presentation

### 2.1. Personal History

A 64-year-old male patient was admitted to the Emergency Department of the Clinical County Emergency Hospital of Braila in June 2024. He presented a clinical picture suggestive of chronic cholecystitis. He reported experiencing persistent, colic pain in the right hypochondrium and epigastrium, which had been present for over three months, without any abrupt exacerbations. Associated symptoms included longstanding nausea with intermittent bilious vomiting. He also noted gradual, unintentional, moderate weight loss over the same period, alongside generalized malaise, fatigue, and recurrent bloating, with abdominal discomfort. Prior to admission, the patient had one brief episode of low-grade fever.

### 2.2. Clinical Assessment

During the general examination in the Emergency Department, the patient was questioned in accordance with a detailed anamnesis and denied any relevant family history. The physical examination revealed tenderness in the right upper quadrant and a positive Murphy sign, both of which are consistent with a gallbladder condition. The patient presented a body mass index in the normal range, with stable vital parameters.

### 2.3. Laboratory Results

The laboratory results were consistent with mild iron deficiency anemia, with values of hemoglobin of 10.9 g/ dL (normal range 13.8–17.2 g/ dL), hematocrit of 32.1% (normal range 36–44.3%), a low erythrocyte count of 4.2 mil/mcL (normal range 4.7–6.1 mil/mcL), a low mean cellular volume/MCV of 56 fl (normal range 80–100 fl), a low mean corpuscular hemoglobin/MCH of 20 pg (normal range 27–33 pg), and a low corpuscular hemoglobin concentration/CMHC of 20 pg (normal range 32–36 g/dL). Also observed were low serum iron levels, at 57 μg/dL (normal range 65–176 μg/dL), and high transferrin and a total iron-binding capacity of 520 μg/dL (normal range 240–450 μg/dL).

Laboratory findings also included a high alkaline phosphatase/ALP level, at 179 U/L (normal range 44–147 U/L), and a high alanine aminotransferase/ALT level, at 73 U/L (normal range 4–36 U/L). Total bilirubin showed also a slightly elevated value of 1.4 mg/dL (normal range 0.1–1.2 mg/dL).

### 2.4. Imaging Examination

The ultrasound examination revealed a gallbladder with thickened walls (5 mm) that was smaller than normal and contracted, displaying increased echogenicity of the walls. Additionally, the presence of echogenic material within the gallbladder lumen, representing biliary sludge, was observed. This sonographic profile is consistent with signs of chronic cholecystitis.

### 2.5. Surgical Treatment

Taking into account the symptoms, the physical examination, and the paraclinical results, the final diagnosis was chronic cholecystitis and iron deficiency anemia. An elective laparoscopic cholecystectomy was performed on the patient. The specimen was preserved in a 10% formaldehyde solution and sent to the Department of Pathology for detailed examination.

### 2.6. Gross and Histopathological Assessment

The gallbladder presented no additional alterations apart from wall thickening and the presence of biliary sludge. Its dimensions measured 7/3/2.5 cm, and it was collected according to protocols. The gallbladder mucosa showed only minute focal erosion, with an otherwise normal appearance.

**Figure 1:**
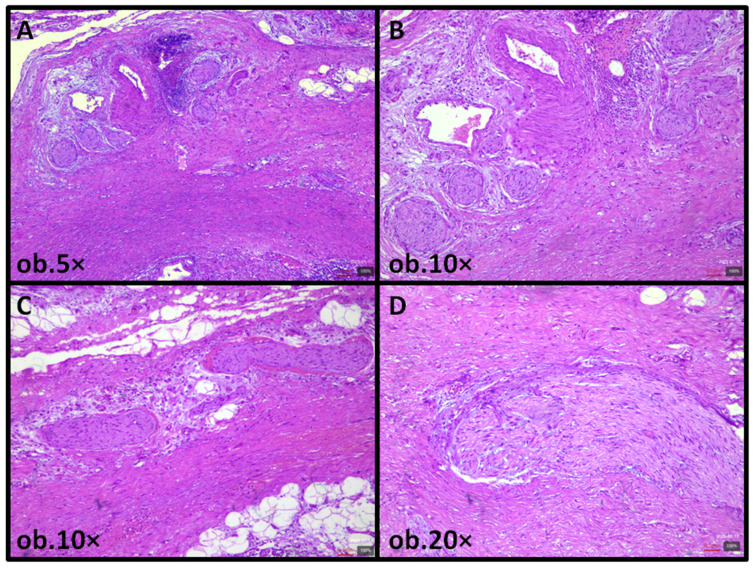
Gallbladder ganglioneuromatosis. Diffuse thickening of the gallbladder showing changes within the fibromuscular layer: hypertrophic nerve fibers and scattered ganglion cells can be seen (**A**,**B**), along with focal adipose substitution (**C**). Proliferation of nerve fibers and ganglion cells in the fibromuscular layer and adventitia (**D**).

Microscopic examination revealed sections with focal erosion accompanied by a chronic, mild-to-moderate lymphocytic inflammatory infiltrate. Within the fibromuscular layer, there was a diffuse proliferation of enlarged nerve fibers, composed of Schwann cells forming interlacing fascicles, together with mature ganglion cells embedded in a collagenous stroma. The process extended circumferentially, replacing and expanding the muscular layer. No cytologic atypia or mitotic activity was identified. The serosa was unremarkable, and no epithelial dysplasia or neoplasia was present (Figure 1).

**Figure 2:**
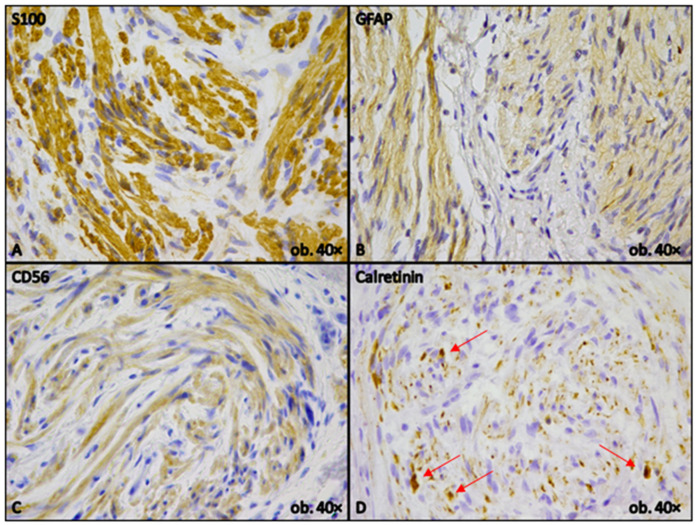
Immunohistochemical profile. Strong, diffuse cytoplasmatic and nuclear positivity for S100 in Schwann cells (**A**). GFAP showing positive cytoplasmic staining in supportive glial cells (**B**). CD56, highlighting neuronal elements, with cytoplasmic staining (**C**). Calretinin nuclear–cytoplasmic positivity for ganglion cells and nerve fibers (red arrows, (**D**)).

Immunohistochemical staining demonstrated positivity for S100 protein, glial fibrillary acid protein (GFAP), Calretinin, and CD56, indicating the presence of hypertrophic nerve fibers and supportive glial-like cells within the gallbladder wall (Figure 2).

### 2.7. Outcome and Follow-Up

The patient was informed of the results and referred to an endocrinologist for the evaluation of the endocrine gland function, as well to a geneticist for the testing of *RET*, *PTEN*, and *NF1* genes. No genetic abnormalities were found.

## 3. Discussion

### 3.1. Published Cases

To contextualize the rarity of our findings, we performed an extensive search across PubMed, Scopus, and Web of Science. This search identified five previously published cases of neurogenic gallbladder lesion. All of them represented true diffuse gallbladder ganglioneuromatosis, one of them without any syndromic association [4,5,6,7,8]. In addition, two cases of solitary polypoid ganglioneuroma of the gallbladder have been published, representing a localized lesion rather than a diffuse mural involvement. Although a distinct from ganglioneuromatosis, this case demonstrates that neurogenic tumors of the gallbladder, in any form, are exceedingly uncommon [9,10]. Beyond the biliary system, our search identified multiple reports of intestinal ganglioneuromatosis, a spectrum that includes solitary ganglioneuroma, ganglioneuromatous polyposis, and diffuse intestinal ganglioneuromatosis [11,12,13,14]. These forms are considerably more common than their gallbladder counterpart and occur predominantly in pediatric and young adult populations. Published cases have been documented in children as young as 1–9 years of age. The typical symptoms were nonspecific, such as abdominal pain and altered bowel habits (constipation, diarrhea or, in more extensive forms, intestinal obstruction) [12,13,14,15,16,17].

This overall distribution pattern underscores a key point: although ganglioneuromatosis is well documented in children and young adults, particularly in the intestinal tract, its occurrence in the gallbladder is uncommon, often clinically silent or similar to chronic cholecystitis, and therefore it is unlikely to be recognized before histopathological examination [3,16].

Syndromic association is well documented. Intestinal ganglioneuromatosis has been reported in the context of neurofibromatosis type 1 (*NF1*), often with diffuse or plexiform involvement, Cowden syndrome (mutation in *PTEN* gene), typically with ganglioneuromatous polyposis and multiple endocrine neoplasia type 2B (*RET* mutation), and usually with diffuse gastrointestinal involvement [3,4,7,18,19,20,21,22].

Despite the relatively larger number of intestinal cases, biliary involvement remains exceptionally rare, likely due to the paucity of intrinsic nerve plexus fibers and ganglion cells in the gallbladder (Table 1) [4,5,6,7,8,9,23]. This distinction supports the notion that gallbladder ganglioneuromatosis represents an anatomical and biological outlier within the spectrum of neurogenic proliferations of the gastrointestinal tract.

### 3.2. Differential Diagnosis

Gallbladder ganglioneuromatosis is defined by a diffuse, benign proliferation of neural elements involving the fibromuscular layer. The hallmark diagnostic criteria include hypertrophic, well-organized nerve bundles replacing and expanding the muscular layer, intermixed mature ganglion cells with abundant cytoplasm and proeminent nucleoli, and the presence of Schwannian cells forming interlacing fascicles without cytologic atypia [1,2,24].

Diffuse ganglioneuromatosis of the gallbladder must be distinguished from several benign and neoplastic entities that may exhibit neural differentiation, mural thickening, or stromal spindle cell proliferation. The differential diagnosis includes neurogenic tumors, reactive neural lesions, neuroendocrine proliferations, and non-neural spindle cell lesions. Each entity can be separated by integrating architectural features, cytological criteria, and immunohistochemical profiles [23,24].

**Traumatic neuroma**

Traumatic neuroma is a reactive, non-neoplastic proliferation of a previously injured nerve, most commonly occurring after surgery, amputations, or blunt trauma. Rather than representing a true tumor, it consists of a haphazard mixture of regenerating axons, Schwann cells, fibroblasts, and dense scar tissue, forming a nodular or fusiform lesion at the site of nerve damage.

Traumatic neuroma can be excluded based on the presence of ganglion cells, the diffuse mural patterns, and the absence of any prior biliary surgery of trauma [25,26].

**Neurofibroma**

For an exhaustive differential diagnosis, neurofibroma should be considered, which is a benign neurogenic tumor composed of an admixture of Schwann cells, fibroblasts, mast cells, and entrapped axons within a characteristic myxoid “shredded carrot” stroma. Lesions are typically poorly circumscribed and infiltrative, occasionally containing scattered, entrapped ganglion-appearing cells, but without a fascicular architecture. In our case, the stroma is not myxoid or shredded, the fascicles are orderly and diffusely thickened, and mature ganglion cells are present. The absence of plexiform growth and the lack of neurofibromatosis type 1-associated features exclude neurofibroma in favor of diffuse ganglioneuromatosis [2,27].

**Neuronal dysplasia (ND)**

ND is a congenital abnormality of the enteric nervous system involving more commonly the intestine (IND) characterized by hyperplasia of submucosal ganglion cells, enlarged ganglia, and the clustering of immature ganglion cells. The proliferation is mucosal or submucosal, not transmural, and occurs almost exclusively in infants and young children, typically presenting with severe constipation or pseudo-obstruction. This pattern is incompatible with our findings, which point us to ganglioneuromatosis [28,29].

**Crohn’s disease**

Crohn’s disease may be considered in the differential diagnosis because it can produce mural thickening, submucosal nodularity, and luminal narrowing, findings that overlap radiologically with ganglioneuromatous proliferations. At the microscopic level, however, Crohn’s disease shows transmural chronic inflammation, granulomas, fissuring ulcers, and reactive neural hyperplasia. Mature ganglion cells are absent, and the nerve bundles are irregular, reactive, and accompanied by active or chronic inflammatory changes [30].

**Schwannoma**

Schwannoma is a benign peripheral nerve sheath tumor composed exclusively of Schwann cells, typically forming a well-circumscribed or encapsulated spindle cell proliferation with Antoni A and Antoni B areas, Verocay bodies, and diffuse S100 immunoreactivity. The lesion lacks mature ganglion cells and demonstrates minimal or absent axonal structures, with neurofilament staining being either negative or limited to entrapped mural proliferations, and their architecture does not expand or replace intrinsic neural plexuses. This pattern is incompatible with our findings, which demonstrate diffuse plexiform proliferations containing mature ganglion cells, Schwannian stroma, and intermingled axonal elements, which are features diagnostic of ganglioneuromatosis [8,31].

### 3.3. Clinical Relevance

Although benign, gallbladder ganglioneuromatosis has important clinical implications. Its presentation often overlaps with common biliary disorders such as chronic cholecystitis or gallbladder wall thickening, leading to the potential misinterpretations of imaging studies. Ultrasound findings may be nonspecific, being frequently reported as a contracted or thickened gallbladder, and no imaging modality reliably distinguishes this entity preoperatively. Because symptoms are frequently mild, intermittent, or attributed to coexisting inflammatory changes, the diagnosis is almost always established histologically following cholecystectomy [3,4,5,6,7,8,9].

Recognition of this lesion is essential for two major reasons: First, correct identification prevents misclassification as neural hyperplasia or as other spindle cell proliferation, which could lead to inappropriate clinical assumptions [32]. Second, gallbladder ganglioneuromatosis, particularly when diffuse, is associated with hereditary syndromic features; histological identification should prompt targeted clinical evaluations and genetic counseling. Early recognition of underlying genetic conditions has significant therapeutic and prognostic implications, especially in MEN2B, where early detection of *RET*-driven medullary thyroid carcinoma can be lifesaving [33].

### 3.4. Genetic Background

Diffuse neurogenic proliferations of the gastrointestinal tract have been associated with alterations in pathways regulating neural crest-derived cells. Germline mutations in *RET*, *NF1*, or *PTEN* underlie the syndromic forms of ganglioneuromatosis described in the literature, although most reported cases outline that these syndromes remain sporadic [34]. Our patient exhibited no clinical features suggestive of a hereditary condition, yet identification of a diffuse neural proliferation warrants basic clinical screening for these disorders; beyond this general framework, detailed molecular modeling or network analysis is not applicable.

## 4. Conclusions

Ganglioneuromatosis of the gallbladder is an exceptionally rare histopathological entity, with only a few cases reported in the international literature. Reporting such cases is essential for expanding the current knowledge of the spectrum of neurogenic biliary lesions and for clarifying potential associations with genetic syndromes.

## Figures and Tables

**Table 1:** Cases of gallbladder ganglioneuromatosis reported up to date (2 December 2025).

Case No.	Authors, Years of Publication	Patient’s Gender	Patient’s Age	Type of Lesion	Associated Syndrome
1.	Modi, 2010 [5]	Female	36 years old	Diffuse ganglioneuromatosis of the gallbladder	Non-syndromic
2.	Takahiko S., 2011 [7]	Male	38 years old	Diffuse ganglioneuromatosis of the gallbladder	MEN2B
3.	Emanuele S., 1991 [9]	Female	37 years old	Diffuse ganglioneuromatosis of the gallbladder	MEN2B
4.	R. Chetty, 1993 [6]	Male	40 years old	Diffuse ganglioneuromatosis of the gallbladder	MEN2B
5.	Sidney Card, 2014 [4]	Female	69 years old	Diffuse ganglioneuromatosis of the gallbladder	MEN2B
6.	Vandana U.G., 2011 [9]	Female	42 years old	Ganglioneuroma of the gallbladder	Non-syndromic
7.	Aseeb Rehman, 2015 [10]	Man	38 years old	Ganglioneuroma of the gallbladder	Non-syndromic

## Data Availability

The original contributions presented in this study are included in the article. Further inquiries can be directed to the corresponding author.
